# Real-World Data on the Use of Cyclin-Dependent Kinase 4/6 Inhibitors in Hormone Receptor–Positive/Human Epidermal Growth Factor Receptor 2–Negative Advanced/Metastatic Breast Cancer

**DOI:** 10.14740/wjon2750

**Published:** 2026-05-08

**Authors:** Αntria Savvidou, Anastasia Constantinidou, Lefteris Zacharia, Kyriaki Michailidou, Μaria Zanti, Stavroula Kitiri, Yiola Marcou, Ifigenia Konstantinou, Eleni Kakouri, Myria Galazi, Christos Petrou

**Affiliations:** a Department of Health Sciences, School of Life and Health Sciences, University of Nicosia, Nicosia, Cyprus; b Department of Medical Oncology, Bank of Cyprus Oncology Centre, Nicosia, Cyprus; c Medical School, University of Cyprus, Nicosia, Cyprus; d Biostatistics Unit, The Cyprus Institute of Neurology and Genetics, Nicosia, Cyprus; e The Breast Center of Cyprus, Nicosia, Cyprus

**Keywords:** CDK4/6 inhibitors, Palbociclib, Ribociclib, Abemaciclib, Metastatic breast cancer, Real-world data

## Abstract

**Background:**

Cyclin-dependent kinase (CDK)4/6 inhibitors (CDK4/6i) have been shown to improve the outcome of patients with hormone receptor–positive (HR+) human epidermal growth factor receptor 2–negative (HER2–) advanced/metastatic breast cancer (a/mBC). This study aimed to demonstrate the safety and effectiveness of CDK4/6i in HR+/HER2- a/mBC patients, in a single-center study in Cyprus and to confirm whether these are in line with data derived from randomized clinical trials and other real-world data studies.

**Methods:**

This retrospective study included 269 patients treated with endocrine therapy (ΕΤ) combined with palbociclib or ribociclib or abemaciclib as first-, second- or third-line treatment at the Bank of Cyprus Oncology Center (2018–2021). Seventy patients from the retrospective study who continued to receive treatment with CDK4/6i-containing regimens, were enrolled in the prospective study (follow-up period: 2021–2024) whereby patients were monitored in real time. Statistical evaluation was performed for differences in progression-free survival (PFS), overall survival (OS), adverse events (AEs)/toxicity, and prognostic factors for effectiveness.

**Results:**

The majority of patients received CDK4/6i in the first-line setting (68%, n = 182/269), whilst 25% and 7.8% of patients received CDK4/6i as second and third line of treatment, respectively. The median progression-free survival (mPFS) was 31, 25 and 19 months, with median overall survival (mOS) of 60, 54 and 44 months for palbociclib, ribociclib and abemaciclib, respectively. Neutropenia was the most commonly reported AE, followed by diarrhea, alanine aminotransferase (ALT)/aspartate aminotransferase (AST) increase, pneumonia, thrombocytopenia, erythematous rash and prolonged QT interval. Following subgroup analyses, age > 65 years, higher BC grade, *de novo* metastatic cancer at initial diagnosis, presence of lymph node/liver or brain metastasis and the larger number of metastatic sites at time of CDK4/6i treatment and the later-line use of CDK4/6i, were associated with a significantly shorter mPFS/mOS.

**Conclusions:**

The combination of CDK4/6i with ET is the gold standard treatment in HR+/HER2– a/mBC. Our study results confirm the effectiveness and tolerability of CDK4/6i in clinical routine practice with prognostic factors aligning with those identified in previous studies. This is the first real–world data (RWD) describing the effectiveness and toxicity of three CDK4/6i in the Cypriot patients.

## Introduction

Breast cancer (BC) is the most commonly diagnosed type of cancer in the world, with more than 2.3 million new BC cases registered every year and almost 685,000 BC deaths worldwide [1]. The majority of patients (70%) with BC are hormone receptor–positive (HR+), and human epidermal growth factor receptor–negative (HER2–) [2]. Treatment options for patients with HR+/HER2– advanced/metastatic BC (a/mBC) have changed over the past decade with the introduction of targeted, personalized treatment options [3]. Inhibition of cyclin-dependent kinase (CDK)4 and CDK6 has shown considerable promise in attenuating resistance to endocrine therapy (ET). CDK4 and CDK6 are not only essential for G1 to S phase cell cycle transition but also play a central role in the growth of HR+ BC cells [3].

The successful completion of phase II and III randomized controlled trials (RCTs) of the three selective CDK4/6 inhibitors (CDK4/6i) in combination with ET (ET-aromatase inhibitors (AIs) or fulvestrant), has led to the granting of marketing authorization for CDK inhibitors by the European Medicines Agency (EMA) and the Food and Drug Administration (FDA). Palbociclib was evaluated in four registrational trials: PALOMA-1/TRIO-18, PALOMA-1, PALOMA-2 and PALOMA-3. Ribociclib was evaluated in MONALEESA-2, MONALEESA-3 and MONALEESA-7, and abemaciclib was evaluated in MONARCH 1, MONARCH 2 and MONARCH 3. The combination of CDK4/6i + ET is established in the first-line treatment in the metastatic setting, overcoming the resistance observed with traditional ET for the treatment of locally advanced or metastatic HR+/HER2– premenopausal or postmenopausal BC [4]. In addition, these RCTs and subsequent meta-analyses collectively demonstrated that, as compared to ET alone, the addition of CDK4/6i increases progression-free survival (PFS) and overall survival (OS) in patients with HR+/HER2– a/mBC [5].

To date, no RCT has directly compared the safety and efficacy of these three agents head-to-head. In addition, RCTs usually include a highly selected population treated in a controlled environment in order to achieve a better balance of known confounders and as a result, might not necessarily reflect real-world clinical practice. Therefore, real-world data (RWD) emerge as valuable data to better understand how these treatments perform in a non-selected population and to determine whether these agents exhibit similar benefits in routine clinical practice. Accumulation of RWD may also help in defining the longer-term effect of CDK4/6i therapy on organ function reserve, particularly considering the chronic nature of the toxicity and the required management [5].

The objective of this RWD study is the evaluation of the impact of CDK4/6i administration in combination with hormonal therapy in the treatment of HR+/HER2– a/mBC, in the Cypriot population, in terms of life expectancy, patient quality of life and safety. This is the first ever study to document RWD in HR+/HER2– a/mBC in Cyprus, and it incorporates the vast majority of patients treated in the country since the first CDK4/6i became available in 2018.

## Materials and Methods

### Study design and cohort definition

A total of 269 patients with HR+/HER2– a/mBC treated with ET in combination with palbociclib, ribociclib or abemaciclib were retrospectively identified in the Bank of Cyprus Oncology Center (BOCOC) from January 2018 to January 2021. Among these, 70 patients from the retrospective study who continued to receive treatment with CDK4/6i-containing regimens, were enrolled in the prospective study (follow-up period: 2021–2024) whereby patients were monitored in real time. The study was conducted in accordance with the Declaration of Helsinki and approved by the Cyprus National Bioethics Committee (protocol code: ΕΕΒΚ/ΕΠ/2020/57 and date of approval: November 4, 2020).

Inclusion criteria were adult patients (18 years and older) with HR+/HER2– a/mBC, patients who had received CDK4/6i for the treatment of HR+/HER2– a/mBC, and patients who had sufficient clinical, laboratory, imaging and medication data. Exclusion criteria were patients (< 18 years old), patients who did not receive CDK4/6i for the treatment of HR+/HER2– a/mBC, and patients who had insufficient clinical, laboratory, imaging and medication data.

The following fields were recorded for each patient: 1) at initial diagnosis: age, BC orientation, BC subtype, BC grade, and BC stage; 2) at time of CDK4/6i treatment: menopausal status, Eastern Cooperative Oncology Group (ECOG) performance status, number of metastatic sites, line of CDK4/6i therapy, line of ET, ET given in combination with CDK4/6i, starting total daily dose of CDK4/6i, ending total daily dose of CDK4/6i, time to CDK4/6i dose reduction, time to CDK4/6i discontinuation, reasons for CDK4/6i dose reduction and discontinuation, adverse events (AEs) monitored and classified according to the Common Terminology Criteria for Adverse Events (CTCAE) (version 5.0).

Treatment was continued until disease progression or unacceptable toxicity. Palbociclib was administered orally 125 mg per day, on a 3-weeks-on/1-week-off schedule (3/1), along with ET. The first dose reduction was to 100 mg/day, and the second dose reduction was to 75 mg/day. Ribociclib was administered orally 600 mg (three tablets of 200 mg) per day, on a 3-weeks-on/1-week-off schedule, plus ET. The first dose reduction was to 400 mg/day (two tablets), and the second decrease was to 200 mg/day (one tablet). Abemaciclib was administered on a continuous schedule (150 mg, twice daily) plus nonsteroidal AI. The first dose reduction was to 100 mg, twice daily, and the second decreased to 50 mg twice daily. Dose modifications of CDK4/6i were made to manage AEs. No dose modifications were documented for ET.

The study outcome measures was the evaluation of the effectiveness of CDK4/6i therapy, the recording and evaluation of time to dose reduction and time to treatment discontinuation of CDK4/6i due to toxicity or disease progression, the recording and evaluation of AEs/toxicity occurring during CDK4/6i treatment, including their incidence and severity in patients with HR+/HER2– a/mBC in Cyprus and assessing factors such as age, BC stage at initial diagnosis, number of metastatic sites, line of treatment, ECOG at the time of CDK4/6i administration that may influence PFS and OS.

### Statistical analysis

All statistical analyses were performed using the R (v4.3.1) statistical computing language. Demographic, disease and treatment characteristics were summarized with descriptive statistics. Continuous variables were summarized as mean ± standard deviation (SD, range) and/or median (interquartile range). Categorical variables were summarized as count (percentage). Clinical and demographic characteristics were compared between groups using the Kruskal-Wallis rank sum test/analysis of variance (ANOVA) for continuous variables and Chi-square/Fisher’s exact test for categorical variables, following normality checks. Follow-up time ended at the time of CDK discontinuation/disease progression, death or last follow-up. The main outcomes evaluated were the PFS, and OS. PFS was defined as the time from treatment initiation with CDK4/6i-containing regimens until disease progression, death or last follow-up. OS was defined as the time from treatment initiation with CDK4/6i-containing regimens until death or last follow-up. Survival over time was calculated using the Kaplan-Meier estimate, and it was compared between groups using the log-rank test. Univariate and multivariate analyses to assess the prognostic significance were performed using the Cox-proportional hazard regression model. Quantitative association from Cox regression was expressed as hazard ratio (HR) with 95% confidence intervals (CIs). P values lower than 0.05 were considered to be statistically significant.

## Results

### Study population and clinical characteristics

This retrospective study included a total of 269 patients treated with CDK4/6i from 2018 to 2021. Out of the 269 patients, 149 (55.4%) received palbociclib, 103 (38.3%) ribociclib, and 17 (6.3%) abemaciclib (Table 1). Seventy patients from the retrospective study who continued to receive treatment with CDK4/6i-containing regimens from 2021 (follow-up period: 2021–2024) participated in the prospective study allowing the researchers a real time documentation of events particularly of toxicity.

**Table 1:** Clinical Characteristics of Patients With HR+/HER2– a/mBC Receiving CDK4/6i-Containing Regimens

Characteristic^a^	Overall (n = 269)	Palbociclib (n = 149)	Ribociclib (n = 103)	Abemaciclib (n = 17)	P value^b^
Age (years) at initial diagnosis^c^	51 ± 11 (29–82)	49 ± 9 (30–75)	55 ± 13 (29–82)	51 ± 12 (35–70)	0.002
Breast cancer orientation at initial diagnosis					0.4
Left breast	177 (66%)	102 (68%)	66 (64%)	9 (53%)	
Right breast	92 (34%)	47 (32%)	37 (36%)	8 (47%)	
Breast cancer subtype at initial diagnosis					0.2
Ductal carcinoma	190 (71%)	111 (74%)	66 (64%)	13 (76%)	
Lobular carcinoma	79 (29%)	38 (26%)	37 (36%)	4 (24%)	
Breast cancer grade at initial diagnosis					0.6
1	24 (8.9%)	15 (10%)	8 (7.8%)	1 (5.9%)	
2	145 (54%)	80 (54%)	53 (51%)	12 (71%)	
3	100 (37%)	54 (36%)	42 (41%)	4 (24%)	
Breast cancer stage at initial diagnosis					0.001
Early stage I	23 (8.6%)	3 (2.0%)	17 (17%)	3 (18%)	
Early stage II	112 (42.1%)	79 (53%)	30 (28.9%)	3 (18%)	
Locally advanced stage III	36 (13%)	22 (15%)	9 (8.7%)	5 (29%)	
*De novo* metastatic stage IV	98 (36%)	45 (30%)	47 (46%)	6 (35%)	
Menopausal status at time of CDK4/6i treatment					0.5
Postmenopausal	237 (88%)	134 (90%)	88 (85%)	15 (88%)	
Premenopausal	32 (12%)	15 (10%)	15 (15%)	2 (12%)	
ECOG performance status at time of CDK4/6i treatment					0.002
0	27 (10%)	14 (9%)	11 (10%)	2 (10%)	
1	89 (33%)	47 (32%)	38 (37%)	4 (25%)	
2	78 (29%)	38 (26%)	33 (32%)	7 (41%)	
3	75 (28%)	49 (33%)	22 (21%)	4 (24%)	
Metastatic sites at time of CDK4/6i treatment^d^					
Bones	75 (28%)	94 (63%)	68 (66%)	16 (94%)	0.038
Lungs	115 (43%)	36 (24%)	30 (29%)	9 (53%)	0.043
Lymph nodes	178 (66%)	15 (10%)	26 (25%)	4 (24%)	0.003
Brain	45 (17%)	31 (21%)	23 (22%)	1 (5.9%)	0.3
Liver	55 (20%)	55 (37%)	54 (52%)	6 (35%)	0.041

^a^N (%), unless otherwise specified. ^b^Kruskal-Wallis rank sum test; Fisher’s exact test. ^c^Mean ± SD (range). ^d^Patients can have multiple metastatic sites. ECOG: Eastern Cooperative Oncology Group; SD: standard deviation; a/mBC: advanced/metastatic breast cancer; CDK4/6i: cyclin-dependent kinase 4 and 6 inhibitors; HER2–: human epidermal growth factor receptor 2–negative; HR+: hormone receptor–positive.

#### Initial diagnosis of BC

The average age at initial BC diagnosis was 51 years (range, 29–82 years). More than half of the patients (66%) had cancer in the left breast and 34% in the right breast. In addition, 190 (71%) patients had ductal carcinoma, and 79 (29%) participants had lobular carcinoma. More than half of the patients (54%) had BC grade 2, 100 (37%) grade 3, and 24 (8.9%) grade 1. Furthermore, 135 (50.7%) patients had early stage I and II BC at initial diagnosis, 36 (13%) locally advanced stage III, and 98 (36%) *de novo* metastatic stage IV (Table 1).

#### At time of CDK4/6i administration

Out of the 269 patients, 237 (88%) were postmenopausal and 23 (8.6%) premenopausal. The ECOG performance status at time of CDK4/6i administration was 0 and 1 in 10% and 33%, respectively, and the rest of the patients had ECOG 2 and 3 performance status. Moreover, almost half of participants (48%) had one metastatic site at the time of CDK4/6i administration, 85 (32%) had two metastatic sites, 47 (17%) had three, and seven (2.6%) had four metastatic sites. Of note, 75 (28%) participants had bone metastasis. The majority of study participants had lymph node metastases (66%) at time of CDK4/6i treatment. Also, 43% of participants had lung metastasis, 28% bone metastasis, 20% liver metastasis, and 17% brain metastasis (Table 1).

### Dose modifications and treatment discontinuation

Sixty-eight percent of patients (n = 182/269) received CDK4/6i in the first line; of these, 113 received palbociclib, 61 ribociclib, and eight abemaciclib. According to the product labelling, the majority of patients received initial total daily doses of 125 mg for palbociclib (81%), 300 mg for abemaciclib (82%), and 600 mg for ribociclib (79%). With reference to palbociclib, 28 patients received a daily dose of 100 mg, and one patient received a daily dose of 75 mg. Furthermore, only two patients received a starting daily dose of 200 mg of ribociclib, while 20 patients had a starting daily dose of 400 mg. Only three patients received a 200 mg daily beginning dose of abemaciclib (Table 2).

**Table 2:** Dosing Schedule of CDK4/6i-Containing Regimens

	Overall (n = 269)	Palbociclib (n = 149)	Ribociclib (n = 103)	Abemaciclib (n = 17)	P value^a^
Line of therapy, n (%)					< 0.001
1	182 (68%)	113 (76%)	61 (59%)	8 (47%)	
2	66 (25%)	31 (21%)	32 (31%)	3 (18%)	
3	21 (7.8%)	5 (3.4%)	10 (9.7%)	6 (35%)	
Starting total daily dose of CDK4/6i, n (%)^b^					< 0.001
75 mg	1 (0.4%)	1 (0.7%)	0	0	
100 mg	28 (10%)	28 (19%)	0	0	
125 mg	120 (45%)	120 (81%)	0	0	
150 mg	3 (1.1%)	0	0	3 (18%)	
200 mg	2 (0.7%)	0	2 (1.9%)	0	
300 mg	14 (5.2%)	0	0	14 (82%)	
400 mg	20 (7.4%)	0	20 (19%)	0	
600 mg	81 (30%)	0	81 (79%)	0	< 0.001
Ending total daily dose of CDK4/6i, n (%)^b^					
75 mg	10 (3.7%)	10 (6.7%)	0	0	
100 mg	73 (27%)	73 (49%)	0	0	
125 mg	66 (25%)	66 (44%)	0	0	
150 mg	7 (2.6%)	0	0	7 (41%)	
200 mg	11 (4.1%)	0	11 (11%)	0	
300 mg	10 (3.7%)	0	0	10 (59%)	
400 mg	34 (13%)	0	34 (33%)	0	
600 mg	58 (22%)	0	58 (56%)	0	
Endocrine therapy given in combination with CDK4/6i, n (%)					< 0.001
Fulvestrant	79 (29%)	33 (22%)	36 (35%)	10 (59%)	
Letrozole	190 (71%)	116 (78%)	67 (65%)	7 (41%)	
Line of endocrine therapy in combination with CDK4/6i, n (%)					< 0.001
1	92 (34%)	44 (30%)	43 (45%)	5 (31%)	
2	136 (51%)	98 (66%)	33 (35%)	5 (31%)	
3	31 (12%)	6 (4.1%)	19 (20%)	6 (38%)	
CDK4/6i dose reduction, n (%)	99 (37%)	63 (42%)	32 (31%)	4 (24%)	
Time to CDK4/6i dose reduction (in days)					< 0.001
Number of observations	269	149	103	17	
Mean (SD)	79 (39)	84 (29)	67 (17)	38 (15)	
CDK4/6i dose discontinuation, n (%)	180 (67%)	105 (70%)	69 (67%)	6 (35%)	
Time to CDK4/6i discontinuation (in days)					< 0.001
Number of observations	269	149	103	17	
Mean (SD)	845 (153)	942 (93)	755 (95)	541 (134)	

^a^Kruskal-Wallis rank sum test; Fisher’s exact test. ^b^Daily dose level 1: 125 mg for palbociclib; 600 mg for ribociclib; 300 mg for abemaciclib (FDA-recommended starting daily dose). Daily dose level 2: 100 mg for palbociclib; 400 mg for ribociclib; 150 mg for abemaciclib. Daily dose level 3: 75 mg for palbociclib; 200 mg for ribociclib. CDK4/6i: cyclin-dependent kinase 4/6 inhibitor; SD: standard deviation; FDA: FDA: Food and Drug Administration.

For patients in the palbociclib, ribociclib and abemaciclib cohorts, the average and (SD) time to CDK4/6i dose decrease was 84 (29), 67 (17), 38 (15) days, respectively. Nighty-nine patients (37%) had their dose reduced over the research period as a result of toxicity. These patients were divided into three cohorts: 63 (42%), 32 (28%) and 4 (24%), for palbociclib, ribociclib and abemaciclib, respectively. The average and (SD) time to drug discontinuation for patients in the palbociclib, ribociclib and abemaciclib groups was 942 (93), 755 (95), 541 (134) days, respectively. Throughout the observation period, a total of 180 patients (67%) were discontinued (three patients due to neutropenia and 177 patients due to disease progression), and were divided into three cohorts: those on abemaciclib (n = 6/180), those on palbociclib (n = 105/180) and those on ribociclib (n = 69/180) (Table 2). In terms of ET, letrozole was most frequently administered with palbociclib (78%) and ribociclib (65%), whereas fulvestrant was most frequently prescribed with ribociclib (35%). Furthermore, the first-line (34%), second-line (51%), and third-line (12%) treatment of ET in conjunction with CDK4/6i are provided (Table 2).

### Safety

Neutropenia was the most common reported toxicity event (all-grade: 40.1%, grade 3/4: 24.1%). Other AEs included diarrhea (all-grade: 27.6%, grade 3: 1.5%), followed by alanine aminotransferase (ALT)/aspartate aminotransferase (AST) increase (all-grade: 13.4%, grade 3: 7.8%), pneumonia (all-grade: 8.2%, grade 2: 5.2%), thrombocytopenia (4.8%), erythematous rash (3.3%) and shortness of breath (1.1%). The majority of patients with diarrhea belonged to the abemaciclib cohort, and the majority of patients with neutropenia belonged to the palbociclib and ribociclib cohorts (Table 3).

**Table 3:** Toxicity Occurring in the Cohort of HR+/HER2– a/mBC Patients Receiving CDK4/6i-Containing Regimens

	Overall (n = 269)	Palbociclib (n = 149)	Ribociclib (n = 103)	Abemaciclib (n = 17)	P value^a^
Experienced ≥ 1 protocol-defined AEs, n (%)					
ALT/AST increase grade 1	15 (5.6%)	0	13 (13%)	2 (12%)	6.81 × 10^–6^
ALT/AST increase grade 3	21 (7.8%)	0	21 (20%)	0	2.03 × 10^–9^
Diarrhea grade 1	52 (19%)	38 (26%)	14 (14%)	0	0.005
Diarrhea grade 2	19 (7.1%)	11 (7.4%)	0	8 (47%)	2.52 × 10^–8^
Diarrhea grade 3	4 (1.5%)	0	0	4 (24%)	1.12 × 10^–5^
Neutropenia grade 1	35 (13%)	13 (8.7%)	19 (18%)	3 (18%)	0.048
Neutropenia grade 2	8 (3.0%)	0	8 (7.8%)	0	0.001
Neutropenia grade 3	47 (17%)	44 (30%)	3 (2.9%)	0	6.01 × 10^–9^
Neutropenia grade 4	19 (7.1%)	19 (13%)	0	0	7.35 × 10^–5^
Prolonged QT interval	5 (1.9%)	0	5 (4.9%)	0	0.025
Thrombocytopenia	13 (4.8%)	13 (8.7%)	0	0	0.002
Pneumonia grade 1	8 (3.0%)	0	8 (7.8%)	0	0.001
Pneumonia grade 2	14 (5.2%)	11 (7.4%)	3 (2.9%)	0	0.24
Erythematous rash	9 (3.3%)	0	9 (8.7%)	0	4.32 × 10^–4^
Shortness of breath	3 (1.1%)	3 (2.0%)	0	0	0.4
Dose reduction due to AE, n (%)	99 (37%)	63 (42%)	32 (28%)	4 (24%)	1.89 × 10^–4^
ALT/AST increase grade 3	21 (7.8%)	0	21 (20%)	0	2.03 × 10^–9^
Neutropenia grade 3	47 (17%)	44 (30%)	3 (2.9%)	0	6.01 × 10^–9^
Neutropenia grade 4	19 (7.1%)	19 (13%)	0	0	7.35 × 10^–5^
Prolonged QT interval	5 (1.9%)	0	5 (4.9%)	0	0.025
Pneumonia grade 2	3 (1.2%)	1 (0.6%)	2 (1.9%)	0	0.24
Diarrhea grade 3	4 (1.6%)	0	0	4 (24%)	1.6 × 10^–5^
CDK4/6i discontinuation due to AE, n (%)					
Neutropenia grade 4	3 (1.1%)	3 (2.0%)	0	0	0.4

Follow-up time stopped at the time of CDK discontinuation or end of the study period, whichever came first. ^a^Fisher’s exact test. AE: adverse event; a/mBC: advanced/metastatic breast cancer; CDK4/6i: cyclin-dependent kinase 4 and 6 inhibitors; HER2–: human epidermal growth factor receptor 2–negative; HR+: hormone receptor–positive; AST: aspartate aminotransferase; ALT: alanine aminotransferase.

Toxicities resulted in a dose reduction for 99 (37%) patients in total (palbociclib, 42%; abemaciclib 24%; ribociclib 32%). Specifically, dose reduction for palbociclib was initiated due to neutropenia grade 3/4 (30%/13%), and pneumonia grade 2 (0.6%). Furthermore, dose reduction for ribociclib was initiated due to prolonged QT interval (4.9%), ALT/AST increase grade 3 (20%), pneumonia grade 2 (1.9%) and neutropenia grade 3 (2.9%). Finally, dose reduction of abemaciclib was due to diarrhea grade 3 (24%). In addition, treatment was permanently discontinued due to neutropenia grade 4 in three patients receiving palbociclib (1.1% overall) (Table 3).

### Effectiveness

#### Univariate and multivariate Cox-proportional hazards model for median progression-free survival (mPFS) of patients receiving CDK4/6i

**Figure 1:**
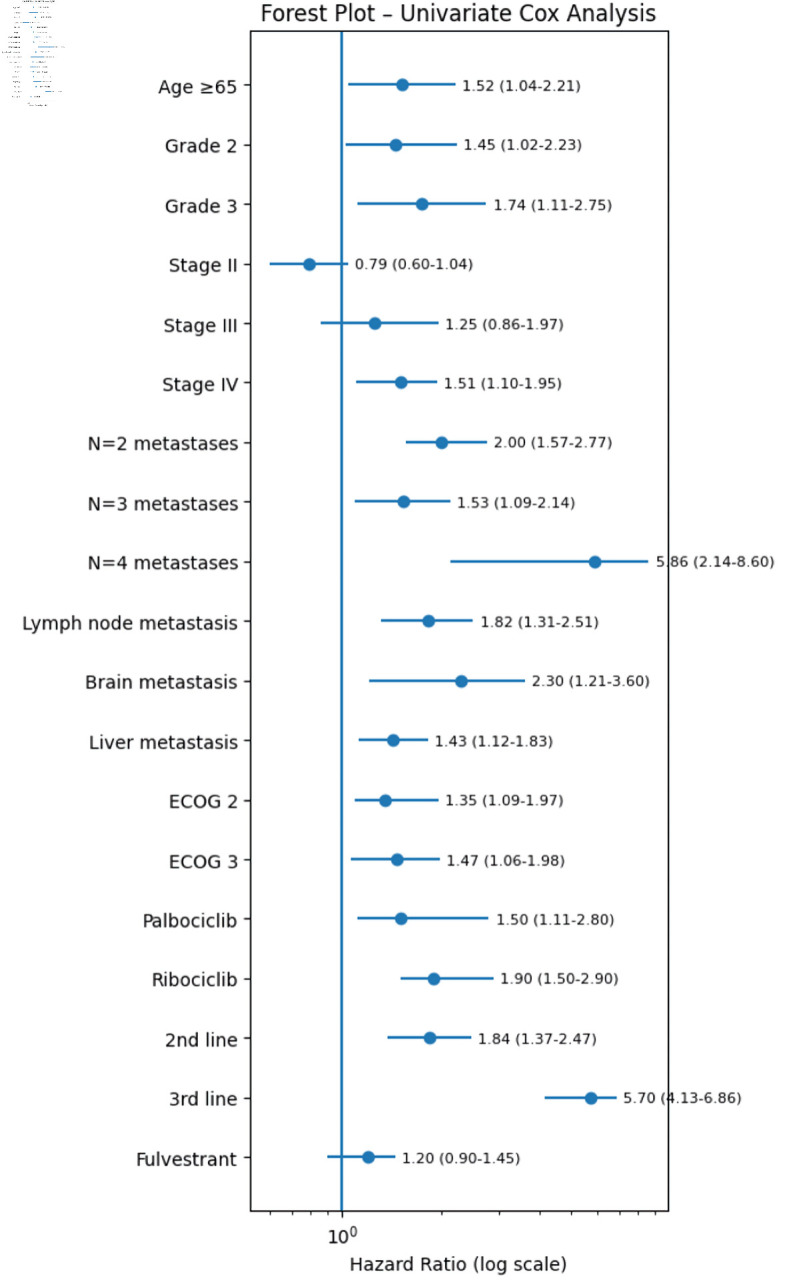
Univariate forest plot model for mPFS of patients receiving CDK4/6i. CDK4/6i: cyclin-dependent kinase 4 and 6 inhibitors; ECOG: Eastern Cooperative Oncology Group; PFS: progression-free survival.

Factors that may influence PFS (mPFS) were examined. Univariate analysis of PFS for the use of CDK4/6i showed that an age of ≥ 65 years, the higher BC grade, *de novo* metastatic stage IV at initial diagnosis, presence of lymph nodes, liver or brain metastasis at time of CDK4/6i treatment, the larger number of metastatic sites at time of CDK4/6i treatment, the higher ECOG at time of CDK4/6i treatment, the use of CDK4/6i in a later line of treatment, and the administration of abemaciclib in contrast to administration of palbociclib or ribociclib, were associated with a significantly shorter mPFS (Table 4). Specifically, in relation to the CDK4/6-related survival, mPFS was 31.5 months for patients who were treated with CDK4/6i in the first line of mBC setting versus 26.3 months and 21.27 months for patients who were treated with CDK4/6i as second- and third-line treatment (HR (95% CI): 1.84 (1.37–2.47), P value = 5.29 × 10^–5^) and (HR (95% CI): 5.7 (4.13–6.86), P value = 1.34 × 10^–4^), respectively (Table 4). Finally, mPFS was 20.27 months for patients who were treated with abemaciclib versus 31.43 months and 25.37 months for patients who were treated with palbociclib and ribociclib (HR (95% CI): 0.02 (0.01–0.8), P value = 4.96 × 10^–8^) and (HR (95% CI): 0.09 (0.05–0.9), P value = 7.59 × 10^–5^), respectively (Table 4, Fig. 1).

**Table 4:** Univariate and Multivariate Cox-Proportional Hazards Model for Median PFS of Patients Receiving CDK4/6i

Univariate analysis						Multivariate analysis		
Reference	No reference	Median PFS (months)	HR	95% CI	P value	HR	95% CI	P value
Age at diagnosis (ref: < 65 years)	≥ 65 years	29.37 vs 25.4	1.52	1.04–2.21	0.03	1.49	1.15–2.54	0.022
Menopausal status (ref: postmenopause)	Premenopause	28.43 vs 26.37	1.01	0.65–1.57	0.96	-	-	-
Breast cancer histology at initial diagnosis (ref: ductal carcinoma)	Lobular carcinoma	29.37 vs 27.4	0.94	0.72–1.23	0.67	-	-	-
Breast cancer grade at initial diagnosis (ref: grade 1)	Grade 2	31.43 vs 28.33	1.45	1.02–2.23	0.04	1.06	0.7–1.9	0.8
	Grade 3	31.43 vs 26.45	1.74	1.11–2.75	0.016	1.52	1.15–2.28	0.032
Breast cancer stage at initial diagnosis (ref: stage I)	Stage II	31.4 vs 29.5	0.79	0.6–1.04	0.1	-	-	-
	Stage III	31.4 vs 27.2	1.25	0.86–1.97	0.42	-	-	-
	*De novo* metastatic stage IV	31.4 vs 26.4	1.51	1.1–1.95	7.38 × 10^–5^	1.87	1.18–2.9	0.04
Number of metastatic sites at time of CDK4/6i treatment (ref: n = 1)	N = 2	31.42 vs 27.37	2	1.57–2.77	4.51 × 10^–3^	1.4	0.6–4.07	0.52
	N = 3	31.42 vs 26.33	1.53	1.09–2.14	0.014	1.9	1.23–4.03	0.059
	N = 4	31.42 vs 23.30	5.86	2.14–8.6	1.41 × 10^–6^	2.35	1.37–5.61	0.045
Bone metastasis (ref: no)	Yes	30.4 vs 26.37	1.16	0.9–1.49	0.26	-	-	-
Lung metastasis (ref: no)	Yes	29.38 vs 27.33	1.17	0.89–1.55	0.25	-	-	-
Lymph node metastasis (ref: no)	Yes	30.37 vs 25.33	1.82	1.31–2.51	3.17 × 10^–4^	1.76	1.14–4.03	0.033
Brain metastasis (ref: no)	Yes	31.8 vs 23.10	2.3	1.21–3.6	0.02	2.1	0.9–2.19	0.175
Liver metastasis (ref: no)	Yes	30.47 vs 26.37	1.43	1.12–1.83	4.09 × 10^–3^	1.32	0.8–2.94	0.2
ECOG performance at time of CDK4/6i treatment (ref: ECOG 1)	ECOG 2	31.4 vs 29.9	1.35	1.09–1.97	0.04	1.18	0.71–2.46	0.73
	ECOG 3	31.4 vs 27.4	1.47	1.06–1.98	0.011	1.25	1.06–1.97	3.09 × 10^–3^
CDK4/6i (ref: abemaciclib)	Palbociclib	20.27 vs 31.43	1.5	1.11–2.8	4.96 × 10^–8^	0.8	0.43–0.9	2.7 × 10^–4^
	Ribociclib	20.27 vs 25.37	1.9	1.5–2.9	7.59 × 10^–5^	0.92	0.62–1.67	0.48
Line of CDK4/6i treatment (ref: first line)	Second line	31.5 vs 26.3	1.84	1.37–2.47	5.29 × 10^–5^	1.17	1.08–3.46	2.39 × 10^–3^
	Third line	31.5 vs 21.27	5.7	4.13–6.86	1.34 × 10^–4^	5.38	2.04–7.08	8.06 × 10^–3^
Partner drug–endocrine therapy (ref: letrozole)	Fulvestrant	31.4 vs 28.37	1.2	0.9–1.45	0.29	-	-	-

HR: hazard ratio; BC: breast cancer; CDK4/6i: cyclin-dependent kinase 4 and 6 inhibitors; CI: confidence interval; ECOG: Eastern Cooperative Oncology Group; PFS: progression-free survival; ref: reference.

On multivariate analysis for mPFS, the administration of CDK4/6i as third line of treatment in contrast to the administration of CDK4/6i as first line of treatment continued to be associated with a significantly shorter mPFS (HR (95% CI): 5.38 (2.04–7.08), P value = 8.06 × 10^–3^) (Table 4). Also, the following predictive factors continued to be associated with a significantly shorter mPFS: 1) *de novo* metastatic stage IV at the initial diagnosis (HR (95% CI): 1.87 (1.18–2.9), P value = 0.04); 2) the larger number of metastatic sites at time of CDK4/6i treatment and specifically three and four metastatic sites vs one metastatic site (HR (95% CI): 1.9 (1.23–4.03), P value = 0.059) and (HR (95% CI): 2.35 (1.37–5.61), P value = 0.045) respectively; 3) lymph node metastasis at time of CDK4/6i administration (HR (95% CI): 1.76 (1.14–4.03), P value = 0.033); 4) ECOG 3 vs ECOG 1 at time of CDK4/6i administration (HR (95% CI): 1.25 (1.06–1.97), P value = 3.09 × 10^–3^); and 5) an age ≥ 65 years in contrast to an age of < 65 years (HR (95% CI): 1.49 (1.15–2.54), P value = 0.022) (Table 4).

The same factors were shown in the prospective arm of the study to be connected with significantly shorter mPFS.

#### Univariate and multivariate Cox-proportional hazards model for mOS of patients receiving CDK4/6i

**Figure 2:**
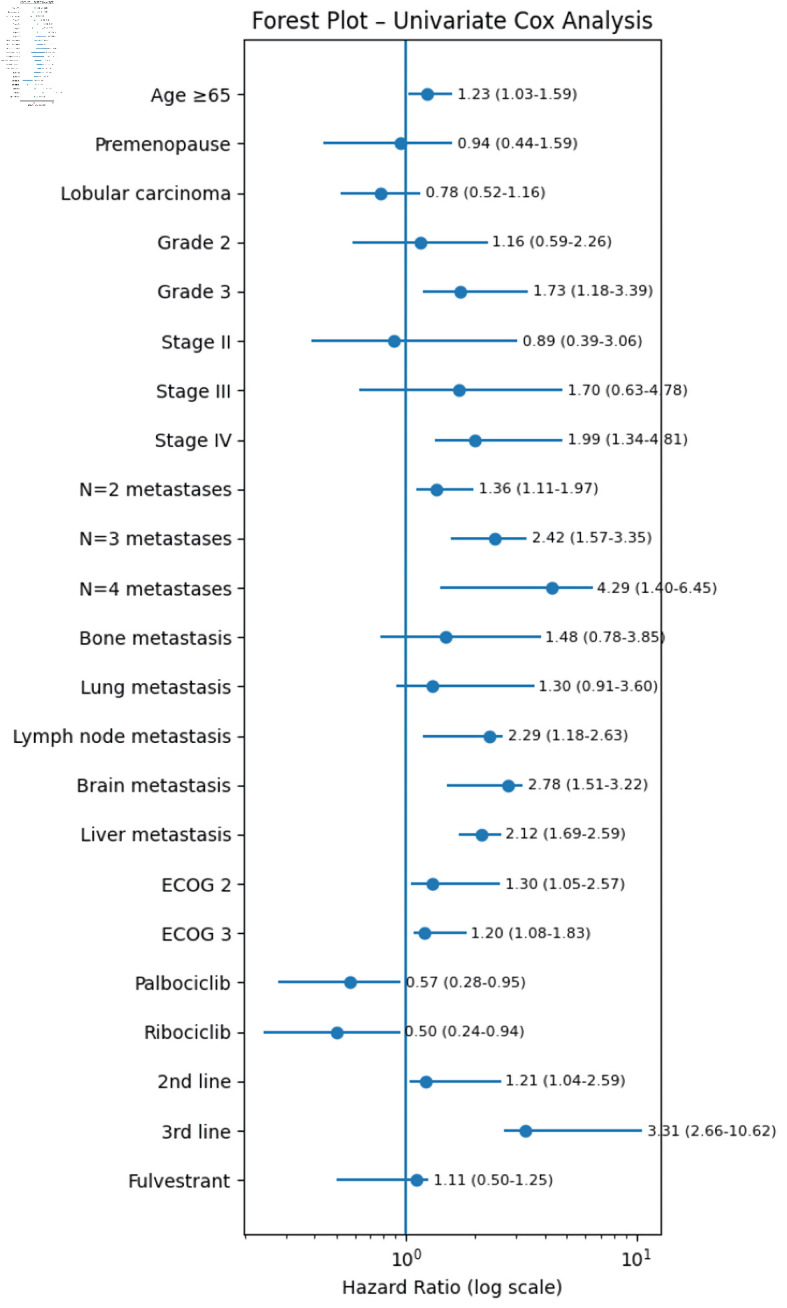
Univariate forest plot model for mOS of patients receiving CDK4/6i. CDK4/6i: cyclin-dependent kinase 4 and 6 inhibitors; ECOG: Eastern Cooperative Oncology Group; mOS: median overall survival.

Factors that may influence OS (mOS) were examined. Median OS was 57.8, 47.43 and 42.67 months in the first line, second line, and third line of treatment and beyond, respectively. Univariate analysis of OS for the use of CDK4/6i showed that an age of ≥ 65 years, the bigger BC grade, *de novo* metastatic stage IV at initial diagnosis, presence of lymph nodes/liver metastasis/brain metastasis at time of CDK4/6i treatment, the bigger number of metastatic sites at time of CDK4/6i treatment, the bigger ECOG at time of CDK4/6i treatment, the use of CDK4/6i in a later line of treatment, and the administration of abemaciclib in contrast to administration of palbociclib or ribociclib, were associated with a significantly shorter mOS (Table 5, Fig. 2).

**Table 5:** Univariate and Multivariate Cox-Proportional Hazards Model for mOS of Patients Receiving CDK4/6i

Univariate analysis						Multivariate Analysis		
Reference	No reference	Median OS (months)	HR	95% CI	P value	HR	95% CI	P value
Age at diagnosis (ref: < 65 years)	≥ 65 years	55.23 vs 51.7	1.23	1.03–1.59	0.042	1.58	1.26–2.69	0.034
Menopausal status (ref: postmenopause)	Premenopause	56.8 vs 53.67	0.94	0.44–1.59	0.68	-	-	-
Breast cancer histology at initial diagnosis (ref: ductal carcinoma)	Lobular carcinoma	55.8 vs 53.7	0.78	0.52–1.16	0.22	-	-	-
Breast cancer grade at initial diagnosis (ref: grade 1)	Grade 2	60.32 vs 55.8	1.16	0.59–2.26	0.66	-	-	-
	Grade 3	60.32 vs 51.2	1.73	1.18–3.39	0.02	1.17	1.03–2.79	0.037
Breast cancer stage at initial diagnosis (ref: stage I)	Stage II	58.83 vs 44.43	0.89	0.39–3.06	0.23			
	Stage III	58.83 vs 40.08	1.7	0.63–4.78	0.36			
	*De novo* metastatic stage IV	58.83 vs 33.4	1.99	1.34–4.81	2.6 × 10^–4^	1.96	1.02–3.4	0.03
Number of metastatic sites at time of CDK4/6i treatment (ref: n = 1)	N = 2	56.73 vs 53.77	1.36	1.11–1.97	0.03	1.28	0.6–1.8	0.73
	N = 3	56.73 vs 50.6	2.42	1.57–3.35	0.6 × 10^–3^	2.15	1.13–4.03	0.02
	N = 4	56.73 vs 46.67	4.29	1.4–6.45	4.29 × 10^–5^	2.56	1.73–6.81	0.039
Bone metastasis (ref: no)	Yes	55.46 vs 50.28	1.48	0.78–3.85	0.12	-	-	-
Lung metastasis (ref: no)	Yes	58.8 vs 51.72	1.3	0.91–3.6	0.29	-	-	-
Lymph node metastasis (ref: no)	Yes	60.77 vs 50.73	2.29	1.18–2.63	0.003	1.53	1.28–3.06	0.017
Brain metastasis (ref: no)	Yes	57.4 vs 48.67	2.78	1.51–3.22	0.001	2.1	1.34–2.19	0.04
Liver metastasis (ref: no)	Yes	56.8 vs 53.73	2.12	1.69–2.59	1.6 × 10^–3^	1.32	0.8–2.94	0.36
ECOG performance at time of CDK4/6i treatment (ref: ECOG 1)	ECOG 2	62.87 vs 54.77	1.3	1.05 – 2.57	0.008	2.07	0.63–4.82	0.39
	ECOG 3	62.87 vs 50.68	1.2	1.08–1.83	0.042	1.39	1.06–1.99	3.1 × 10^–3^
CDK4/6i (ref: abemaciclib)	Palbociclib	43.63 vs 59.77	0.57	0.28–0.95	0.01	0.37	0.2–0.8	2.35 × 10^–5^
	Ribociclib	43.63 vs 53.73	0.5	0.24–0.94	0.04	0.75	0.46–2.93	0.61
Line of CDK4/6i treatment (ref: first line)	Second line	57.8 vs 47.43	1.21	1.04–2.59	0.0033	2.79	1.98–7.36	0.81 × 10^–3^
	Third line	57.8 vs 42.67	3.31	2.66–10.62	2.62 × 10^–4^	4.21	2.01–9.08	1.04 × 10^–4^
Partner drug–endocrine therapy (ref: letrozole)	Fulvestrant	58.33 vs 52.6	1.11	0.5–1.25	0.37	-	-	-

HR: hazard ratio; BC: breast cancer; CDK4/6i: cyclin-dependent kinase 4 and 6 inhibitors; CI: confidence interval; ECOG: Eastern Cooperative Oncology Group; mOS: median overall survival; ref: reference.

The same predictive factors appeared in the multivariate analysis with significantly shorter mOS as previously reported in the multivariate analysis for mPFS (Table 5).

## Discussion

This is the first RWD study documenting the effectiveness and toxicity of three CDK4/6i in HR+/HER2– a/mBC patients in Cyprus. The study includes a total of 269 patients treated with CDK4/6i. The clinical characteristics of this study population were heterogenous across subgroups.

The number of patients treated with each of the three CKD4/6i differs in the study, with palbociclib, the first agent to be FDA and EMA approved, being received by the highest proportion of patients (55.39%), abemaciclib by the lowest (6.32%), and ribociclib with 38.29%. Following their FDA and EMA approvals the three agents became available in the Cypriot market sequentially, with the first being palbociclib in 2018, followed by ribociclib in 2019, and abemaciclib in 2020. Considering that the retrospective study processed patient data between 2018 and 2021, as expected, most patients in the study received palbociclib, a smaller number received ribociclib, and a few patients received abemaciclib [6–11].

The majority of patients in the retrospective study, and by extension also in the prospective study, received CDK4/6i in the first line (68%), whereas 25% and 7.8% of patients received CDK4/6i as second and third line of treatment, respectively. This is documented in other RWD studies, as in the first years of their approval, CDK4/6i were used at different lines of treatment. With the accumulation of efficacy and survival evidence, CDK4/6i consolidated their place in the first-line setting, which is reflected in European and international clinical guidelines [12–16].

Moreover, the association of CDK4/6i with hormone therapy was in accordance with the literature. In line I of treatment, the PALOMA 2 and PALOMA 4 trials paired palbociclib with letrozole, the MONALEESA 2 trial paired ribociclib with letrozole, and the MONARCH 3 trial paired abemaciclib with letrozole. In line II, as shown in PALOMA 3, MONALEESA 3, and MONARCH 2, letrozole was replaced by fulvestrant. In this study, more than half of patients were treated as per this paradigm, with letrozole in the first-line setting and with letrozole or fulvestrant in the second-line setting. Fulvestrant was added to CDK4/6i in a minority of patients in the third-line setting [17–32].

Of note, 35% of patients included in the study were > 65 years of age, with ECOG 2 or 3 (29% and 28% respectively), and with multiple comorbidities, representing a subgroup of patients usually excluded from RCTs. This poses an advantage for the study, which sheds light on data otherwise unavailable for the Cypriot BC population, as highlighted by other previous RWDs in other countries [28, 33]. Therefore, our patient population differs from that of RCTs of CDK4/6i, with patients at higher risk of disease progression [17–32].

Thirty-seven percent and 56% of patients experienced dose reduction due to toxicity retrospectively and prospectively. Percentages of 40–50% dose reduction due to toxicity are generally consistent with RCTs and other RWDs [17–32]. Treatment was definitively discontinued due to disease progression in 67% and 90% of patients in the retrospective and prospective study, respectively. This high percentages of 70–90% of treatment discontinuation due to disease progression are generally consistent with RCTs and other RWDs [17–32]. The inclusion of the prospective phase aimed to ensure high quality data collection, which is not always quarantined with retrospective data; however, on this occasion, it is reassuring that the percentages documented in the retrospective and the prospective phases were similar.

Furthermore, it was noted that dose reduction due to toxicity had no negative impact on PFS during CDK4/6i therapy. It is already known that dose reduction of CDK4/6i due to AEs does not negatively impact clinical outcomes [34]. A possible explanation might be that patients with adjusted reduced dosage need fewer therapy interruptions leading to a constant plasma drug level. According to the above, in one retrospective RWD study, it was observed that patients who required dose reduction had longer PFS compared to patients who did not have a dose reduction [14].

The mean average time to dose reduction was shortest in the abemaciclib cohort, followed by the ribociclib cohort, while the longest was in the palbociclib cohort. This observation is consistent with the management of CDK4/6i in clinical practice, since abemaciclib is more commonly associated with diarrhea therefore requiring immediate dose reduction. Ribociclib is associated with frequent neutropenia and increase in the liver enzymes and prolongation of the QT interval, therefore its dose reduction time due to greater toxicity is shorter than palbociclib [26, 31].

Regarding toxicity in both the retrospective and prospective study population, neutropenia and diarrhea were the most common AEs reported in participants followed by ALT/AST increase, prolonged QT interval, pneumonia, thrombocytopenia, erythematous rash and breathlessness. Specifically in the retrospective study, the majority of patients who experienced neutropenia were in the palbociclib (51.7%) and ribociclib (28.7%) cohorts, whereas the majority of patients who experienced diarrhea were in the abemaciclib cohort (71%). All patients who experienced QT prolongation and the majority of patients who experienced ALT/AST increase were in the ribociclib cohort (33%). These results demonstrated the types of AEs observed in this population were generally consistent with the most frequently reported AEs in RCTs. In the palbociclib and ribociclib RCTs, the most frequently reported all-grade AE was neutropenia (palbociclib treatment arms: 80–81%; ribociclib treatment arms: 74–76%). The most common all-grade AE associated with abemaciclib was diarrhea (85–90%) [17–32].

In other published RWD studies, the toxicity data per agent were generally consistent: neutropenia occurred with all CDK4/6i agents (the highest percentage observed with palbociclib), whilst QTcF prolongation, AST elevation, and ALT elevation were more commonly reported with ribociclib, and diarrhea with abemaciclib. In line with the above, a retrospective study conducted in the UK reported that the majority of patients in the palbociclib cohort (21%) experienced grade 3/4 neutropenia, and in the abemaciclib cohort, they experienced grade 3/4 diarrhea (Buller et al, 2023 [35]). In another retrospective study in Brazil, most patients who received palbociclib experienced neutropenia (63.2%), whilst the majority of patients receiving ribociclib experienced QTcF-prolongation (2.3%), and AST/ALT elevation (16.4%) [12]. The same pattern continued in retrospective RWD in an American study where the majority of patients receiving palbociclib experienced neutropenia (44.8%), and the majority of patients receiving abemaciclib experienced diarrhea (16.4%) [13].

In the retrospective study, the mPFS was 31, 25 and 19 months, and the mOS was 60, 54 and 44 months for palbociclib, ribociclib and abemaciclib, respectively. In the prospective study, the mPFS was 31, 25, and 16.5 months for palbociclib, ribociclib, and abemaciclib, respectively. Although the aim of this study was not to report specifically the mPFS and the mOS, both PFS and OS observed in the study were in line with those in RCTs and other RWDs. The differences in mPFS and mOS in our study are reasonable to observe as the population participating in this retrospective and prospective study was heterogeneous. Specifically, participants received three different CDK4/6i in different lines of treatment, with different combinations of ET, and different clinical characteristics at initial diagnosis and at time of CDK4/6i administration [17–32].

Τhe multivariate subgroup analyses for both retrospective and prospective study showed that patients aged ≥ 65 years, higher BC grade, *de novo* metastatic stage IV at initial diagnosis, a larger number of metastatic sites and higher ECOG at time of CDK4/6i treatment, as well as the use of CDK4/6i in a later line of treatment, were associated with a significantly shorter mPFS.

In our study, the mPFS for palbociclib was longer than the results from the RCTs PALOMA 2 (n = 444) and PALOMA 4 (n = 340), which showed a mPFS of 27.6 months (95% CI, 22.4–30.3) and 21.5 months (95% CI, 16.6–24.9) respectively, for palbociclib plus letrozole; whilst in PALOMA 3 (n = 347), the mPFS achieved with palbociclib plus fulvestrant was 11.2 months (95% CI, 9.2–not reached). It should be noted that PALOMA 2 and PALOMA 4 included patients who received only letrozole with palbociclib, in contrast to PALOMA 3, where patients received only fulvestrant as ET. Furthermore, in PALOMA 2 and PALOMA 4, palbociclib was administered in the first line of treatment only, which is in contrast to this study where patients received palbociclib as first, second and third line of treatment [17, 20, 21]. This is due to the fact that the study group under consideration was quite heterogeneous, thus reflecting that the population of RWD studies differs from the population of RCTs and useful conclusions are drawn for clinical practice.

A RWD study published by Wong et al (2022) described the use of ribociclib plus AI as a first-line treatment for HR+/HER2− mBC in the Australian population. With data from 160 patients, the mPFS was 36.3 months, while in MONALEESA-2, it was 25.3 months [22, 36]. Regarding a more recently published retrospective RWD Romanian study that included three CDK4/6i in all lines of treatment, an overall mPFS of 17 months (95% CI, 14.89–20.32) was documented, which is much lower than the mPFS of 31 months (95% CI, 30–33) in this study. Specifically, with data from 160 patients, mPFS was 22.9 months (95% CI, 18.67–27.12), 12.06 months (95% CI, 9.65–14.47) and 11.07 months (95% CI, 6.41–17.06) for palbociclib, ribociclib and abemaciclib, respectively, in contrast to our study which presented higher mPFS for all three agents: 31 months (95% CI, 30–33), 25 months (95% CI, 23–27) and 19 months (95% CI, 13–22) for palbociclib, ribociclib and abemaciclib, respectively[33]. The reason is that in our study, there is more heterogeneity in prior treatment, patient comorbidities, and treatment partners of CDK4/6i, which complicates the interpretation of the results [33].

In addition, in this study, the first line of CDK4/6i treatment was shown to be associated with a significantly higher mPFS compared to the second or third/subsequent line of treatment. The mPFS was 31 months (95% CI, 27–31), 26 months (95% CI, 23–28) and 21 months (95% CI, 17–22) for first, second and third line of CDK4/6i treatment. Similar patterns were shown in another large retrospective RWD study conducted in Germany (n = 448), where the mPFS was 23 months, 13 months, and 11 months for first, second, and third line of CDK4/6i treatment. Τhe difference between the above study and our study is most likely due to the different population participating in each study. The proportion of patients that received palbociclib, ribociclib and abemaciclib (71.3%, 25.4%, 3.3% vs 55.39%, 38.29%, 6.32% in this retrospective study) was not the same. It is important to note that the proportion of patients that received CDK4/6i as first, second and third line of treatment was also different between the two studies (62.1%, 19.2%, 19.7% vs 68%, 25%, 7.8% in this retrospective study) [14].

The mOS in this population receiving ribociclib (n = 103) was 54 months (95% CI, 40–62). This result was consistent with the mOS achieved in the ribociclib RCTs. Specifically, in MONALEESA 2 (n = 334), the mOS achieved with ribociclib plus letrozole was 63.9 months (95% CI, 52.4–71); in MONALEESA 3 (n = 484), the mOS achieved with ribociclib plus fulvestrant was 53.7 months (95% CI, 46.9–not reached). Also, in MONALEESA 7 (n = 335), the mOS was 58.7 months (HR: 0.73) for ribociclib plus ET (goserelin plus nonsteroidal aromatase inhibitor (NSAI) or tamoxifen). However, it should be noted that MONALEESA 2 and 3 included only postmenopausal patients, MONALEESA 7 included only premenopausal patients and up to 70% of patients had disease recurrence. In this retrospective study, 88% of patients were postmenopausal and 12% were premenopausal. In MOLANEESA 2, patients were treated with ribociclib in the first line only, which was not the case in this retrospective study, where 59%, 31%, 10% patients received ribociclib as first, second and third line of treatment respectively [23, 25, 28].

In this cohort, we observed that patients with abemaciclib (n = 17) achieved mOS of 44 months (95% CI, 33–46). Specifically, in MONARCH 2 (n = 446), the mOS achieved with abemaciclib plus fulvestrant was 46.7 months; in MONARCH 3 (n = 328), the abemaciclib with AI arm had a significantly longer mPFS of 67.1 months. Notably, the sample size of patients treated with abemaciclib in this study was too small to correlate with this series of data and to conclude statistically significant results. In addition, the mOS observed in this study for abemaciclib (20 months) was higher than that observed in the RCT in endocrine-resistant patients (Monarch 2: 16.4 months). It is also noted that, in MONARCH 2, patients received only fulvestrant as ET in combination with abemaciclib, in contrast to this study, where patients received letrozole and fulvestrant plus any of three CDK4/6i [30, 32].

In another RWD study of 217 patients receiving any of the three CDK4/6i in all lines of treatment, mOS was 38 months (95% CI, 33.5–42.5), 33.9 months (95% CI, 26.7–41.1) and 27.3 months (95% CI, 22.5–32.1) for palbociclib, ribociclib and abemaciclib, respectively. In the current study, mOS was longer, at 60 months (95% CI, 44–68), 54 months (95% CI, 40–62) and 44 months (95% CI, 33–46) for palbociclib, ribociclib and abemaciclib respectively. A possible explanation for the difference in mOS between the UK study and this study is the higher proportion of patients with ≥ 3 metastatic sites at the time of CDK4/6i administration in the UK study compared with our study (71% vs 27%) [36].

In this study, patients receiving CDK4/6i-based treatment as first line had a significantly higher mOS compared to those receiving CDK4/6i as second or third/subsequent line of treatment, with mOS of 58 months (95% CI, 45–66), 48 months (95% CI, 31–60) and 43 months (95% CI, 34–58) for first, second and third line of CDK4/6i treatment. In a retrospective RWD from Greece (n = 365), the mOS was not reached, 29.9 months and 20.4 months for first, second and third line of CDK4/6i-based treatment. This may be related to the different percentage of patients receiving each CDK4/6i in each study. The proportion of patients receiving palbociclib, ribociclib and abemaciclib (82.5%, 17.5%, 0% vs. 55.39%, 38.29%, 6.32% in this retrospective study) was not the same [15].

The limitation of the study was that the study group under consideration was quite heterogeneous, but this only reflected the fact that the population of RWD studies differs from the population of RCTs, and useful conclusions are drawn for clinical practice. The retrospective nature of the data created gaps, with overall completeness of the retrospective data being 80%; however, this was addressed by conducting the prospective study in which patients from the retrospective study continued to receive CDK4/6i treatment and were followed for two consecutive years, achieving 100% data completeness.

The study cohort has a very diverse treatment population (i.e., many CDK4/6i and hormone treatments), which may make comparisons between subgroups, especially those with fewer patients, more difficult. Because of the population’s heterogeneity, care should be taken when interpreting the comparisons and findings reported here. Furthermore, fewer patients received abemaciclib treatment than the other two agents, as a result of the FDA’s and EMA’s subsequent approval. Consequently, in terms of PFS, direct comparisons between abemaciclib and the other two treatments would not be reliable. The fact that the study group under examination was quite heterogeneous is a limitation of this study, but it also shows how RWD can differ from RCTs and illustrates the benefit of this analysis. Furthermore, the study of the use of these agents in Cypriots, provides real-life evidence for a small country whose patients do not often participate in large international RCTs.

### Conclusions

The development of CDK4/6i combinations with hormonal agents has been a major advance in the treatment of estrogen-positive a/mBC. The added synergistic activity has been shown to be superior to hormonal agents alone in RCTs phase III, and with the convenience of oral dosing, these regimens have become standard first-line regimens of choice. However, the toxicity and effectiveness of any of the three licensed agents require careful monitoring and management through RWD studies. Their use has increased in a broader population (more reflective of RWD than the RCTs). The long-term effects of CDK4/6i therapy, particularly considering the chronic nature of toxicity, and how this affects outcome with subsequent anticancer therapy, remain an area of ongoing research.

Through the current RWD study, it is shown that the use of CDK4/6i-based treatment in Cypriot patients with HR+/HER2– a/mBC, is safe and effective. The results are in line with results derived from RCTs and other previous RWD studies.

In conclusion, this study compared the three molecules (palbociclib, ribociclib, and abemaciclib) in a population that was more diverse than that included in RCTs and assessed CDK4/6i outcomes in RWD studies. The results of this study not only support a conclusive and comparable assessment of CDK4/6i’s effectiveness, therapeutic benefit, and safety profile, but they also raise the possibility that CDK4/6i may offer results that are similar with or even superior to those reported in RCTs.

This is the first RWD study that provides valuable data from everyday clinical practice for Cypriot patients and compare the three CDK4/6i to each other, bridging the gap between RCTs and clinical reality in Cyprus.

### Learning points

This is the first RWD study to describe the effectiveness and toxicity of three CDK4/6i in the Cypriot patients. Also, this observational clinical study was conducted in special populations, especially in patients > 65 years of age, with multiple comorbidities, and ECOG 2 and ECOG 3, a community that is usually underrepresented or not represented at all in RCTs. The knowledge extracted from this study will be used by oncologists with the aim of better utilizing personalized medication with the least adverse effects, and thereby optimizing the quality of life of patients. In addition, the results of this study can be used by Cypriot, European and international health regulatory bodies to review or update pharmaceutical protocols and drug administration guidelines.

## Data Availability

The data that support the findings of this study are available from the corresponding author upon reasonable request.

## Abbreviations

a/mBC: advanced/metastatic breast cancer
AE: adverse event
AIs: aromatase inhibitors
BOCOC: Bank of Cyprus Oncology Center
BC: breast cancer
CI: confidential interval
CDK4/6i: cyclin-dependent kinase 4 and 6 inhibitors
ECOG: Eastern Cooperative Oncology Group
CTCAE: Common Terminology Criteria for Adverse Events
EMA: European Medicines Agency
ET: endocrine therapy
FDA: Food and Drug Administration
HER2–: human epidermal growth factor receptor 2–negative
HR+: hormone receptor–positive
HR: hazard ratio
mBC: metastatic breast cancer
NA: not applicable
OS: overall survival
PFS: progression-free survival
RCTs: randomized controlled trials
RWD: real-world data
